# FTO4/449: Use of Software Agents for Treating Disease over the Internet

**DOI:** 10.2196/jmir.1.suppl1.e29

**Published:** 1999-09-19

**Authors:** R Leca, E Kettle, L Osvath

**Affiliations:** ^1^Biosign Corporation, RoseneathCanada

**Keywords:** Telemedicine, Biosignal Processing, Mobile Software Agents, Asthma

## Abstract

**Introduction:**

Mobility, responsivity, autonomy, learning, and collaboration are characteristics commonly shared by biological and software agents. Our investigation into the physiological resources of communication suggested the possibility of using software rather than chemical agents in the treatment of disease. Assuming that the human body has adapting host platforms to agents presented in a form coherent with the biological conditions of receptivity, we developed a system that enables software agents to transport their state into the human body. A small-scale feasibility study was conducted to determine the value of this novel research path.

**Methods:**

For a case-comparison, we studied 12 volunteers suffering from mild to moderate asthma and 15 healthy volunteers as controls. The subjects were teleconsulted over the Internet using a custom system (Biosign, Canada) that programs progressive analysis of Pulsewave and Oximetry Parameters (POP) acquired using a DAQ board (National Instruments, USA). POP patterns were identified and interpreted by an expert system integrated between the sensor and the effector units. Software agents were selected based on POP patterns and deployed over the Internet.

**Results:**

All patients exhibited significant POP pattern divagation (P<0.001) compared with the controls. Four patients responded promptly to treatments using software agents, with no relapse during a six-week follow-up. The other patients did not respond at all. No side effects have been observed or reported.

**Discussion:**

The results are discussed for each case. They suggest that digital therapy - namely, the therapeutic use of software agents, may be a viable and desirable alternative, especially when assessed according to the primary aims of telemedicine. Treating disease by means of software agents deployed over the Internet is, in our opinion, a goal attainable within the limits of current technology.

